# Supplementary material for: Air transportation, population density and temperature predict the spread of COVID-19 in Brazil
Source: PeerJ. 2020 Jun 3;8:e9322. doi: 10.7717/peerj.9322 (PMC7275681; doi:10.7717/peerj.9322)
Supplement: Supplemental Information 1 — Results of the complementary analysis using only the 275 observations for which there were complete precipitation data. [file peerj-08-9322-s001.docx]

**Supplementary Material**

**Air transportation, population density and temperature predict the spread of COVID-19 in Brazil**

Pedro Aurélio Costa Lima Pequeno^1^, Bruna Naissinger Mendel^2^, Clarissa Alves da Rosa^3^, Mariane Bosholn^1^, Jorge Luiz Pereira Souza^4^, Fabricio Beggiato Baccaro^5^ , Reinaldo Imbrozio Barbosa^1^, William E. Magnusson^3^,

^1^ Instituto Nacional de Pesquisas da Amazônia, Boa Vista, Roraima, Brazil

^2^ Universidade Federal de Roraima, Boa Vista, Roraima, Brazil

^3^ Instituto Nacional de Pesquisas da Amazônia, Manaus, Amazonas, Brazil

^4^ Instituto Nacional da Mata Atlântica, Santa Teresa, Espírito Santo, Brazil

^5^ Universidade Federal do Amazonas, Manaus, Amazonas, Brazil.

Corresponding Author:

Clarissa Alves da Rosa

Av. André Araújo, 2936, Manaus, Amazonas, CEP 69067-375, Brazil

Email address: rosacla.eco@gmail.com

**Description**

Table S1 is .csv file containing the analyzed daily cumulative counts of confirmed COVID-19 cases by Brazilian capital city (n = 292 daily counts).

Table S2 is a .csv file containing the used geographic, demographic and socioeconomic data for Brazilian capital cities (n = 27 capital cities).

Table S3 is a .scv file containing the raw data on flights arriving in airports within the metropolitan areas of Brazilian capital cities in 2020 (n = 3815 flights).

Table S4 contains the raw hourly data on meteorological variables (n = 73,444 hours).

Figs. S1 and S2, and Table S5 (this file) contain the results of the complementary analysis using only the 269 observations for which there were complete precipitation data.


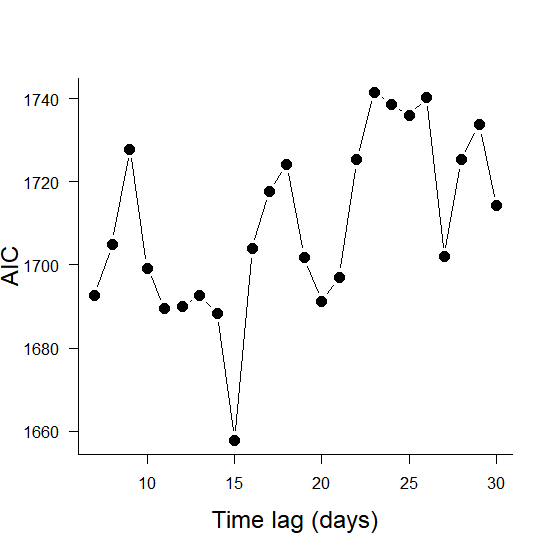


Fig. S1 Comparison of models considering different time lags in meteorological predictors (including mean daily precipitation) using Akaike’s Information Criterion (AIC) (n = 269). Each point represents one alternative model.

Table S5 Results of the most supported Generalized Linear Mixed Model (GLMM) for daily counts of confirmed COVID-19 across 25 capital cities in Brazil, including mean daily precipitation as predictor (n = 269). The model assumed Poisson-distributed errors and log link, and used capital city identity as a random factor to account for autocorrelated errors of time series within cities. All predictors were scaled to zero mean and unit standard deviation. SD indicates predictor standard deviation; numbers in bold represent statistically significant effects (*P* < 0.05). Variables were as follows: time - time elapsed in days; density - inhabitants by km²; elders - number of elders (≥ 60 years old) per 1000 citizens; physicians - number of registered physicians per 1000 citizens; income - mean citizen income (R$); flights - number of arriving flights in 2020; temperature - mean daily temperature (ºC) with a 15-day lag; radiation - mean daily solar radiation (kJ/m²) with a 15-day lag; humidity – mean daily air humidity (%); precipitation – mean daily precipitation (mm) with a 15-day lag.

| Predictor | SD | Coefficient | Z | P |
| --- | --- | --- | --- | --- |
| (Intercept) | - | 2.27 | - | - |
| Time | 5.74 | 1.41 | 67.05 | **<0.001** |
| Log suspected | 1.76 | 0.29 | 0.85 | 0.393 |
| Log flights | 1.22 | 0.84 | 2.9 | **0.004** |
| Density | 3182.49 | 0.46 | 1.85 | 0.065 |
| Elderly | 3.71 | -0.38 | -1.55 | 0.122 |
| Income | 743.11 | 0.08 | 0.39 | 0.698 |
| Temperature | 3.16 | -0.26 | -5.77 | **<0.001** |
| Radiation | 246.34 | 0.03 | 1.23 | 0.218 |
| Humidity | 10.38 | 0.1 | 2.3 | **0.021** |
| Precipitation | 0.75 | -0.05 | -2.42 | **0.016** |


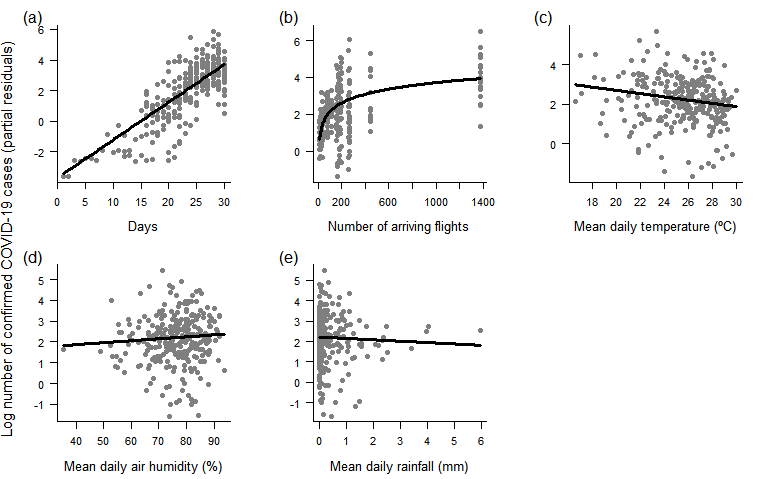


Fig. S2 Response of daily counts of confirmed COVID-19 cases across Brazilian capital cities to (a) time, (b) number of arriving flights, (c) temperature, (d) mean daily air humidity and (e) mean daily rainfall, as indicated by the most supported Generalized Linear Mixed Model (GLMM). The model assumed Poisson-distributed errors and log link, and included capital city identity as a random factor to account for autocorrelated errors in time series within cities. Each point represents a daily observation in a given city (n = 269); lines represent predicted means. Plots use partial residuals of the response variable and thus show the effect of a given predictor while controlling the effects of remaining predictors.
